# Measurement properties of oral health related patient reported outcome measures in patients with oral cancer: A systematic review using COSMIN checklist

**DOI:** 10.1371/journal.pone.0218833

**Published:** 2019-06-27

**Authors:** Shailesh M. Gondivkar, Amol R. Gadbail, Sachin C. Sarode, Rima S. Gondivkar, Monal Yuwanati, Gargi S. Sarode, Shankargouda Patil

**Affiliations:** 1 Department of Oral Medicine & Radiology, Government Dental College & Hospital, Nagpur, Maharashtra, India; 2 Department of Dentistry, Indira Gandhi Government Medical College & Hospital, Nagpur, Maharashtra State, India; 3 Department of Oral Pathology & Microbiology, Dr. D.Y. Patil Dental College & Hospital, Dr. D.Y. Patil Vidyapeeth, Maharashtra State, Pune, India; 4 Independent consultant, Nagpur, Maharashtra State, India; 5 Department of Oral Pathology & Microbiology, Peoples College of Dental Sciences, Bhopal, Madhya Pradesh, India; 6 Department of Maxillofacial Surgery and Diagnostic Sciences, Division of Oral Pathology, College of Dentistry, Jazan University, Jazan, Saudi Arabia; Iranian Institute for Health Sciences Research, ISLAMIC REPUBLIC OF IRAN

## Abstract

**Background:**

Oral cancer (OC) is one of the common malignant neoplasm resulting in a range of debilitating symptoms. Patient reported outcome measures (PROMs) could provide a valuable insight into the impact of OC on patients’ quality of life (QoL). Selecting an adequate instrument among available PROMs for OC has been challenging for clinicians due to lack of information on their psychometric quality. This systematic review provides an extensive overview of methodological quality of all currently available PROMs for OC.

**Method:**

A systematic search was performed in PubMed, Scopus, Web of Science and CINAHL for relevant literature until 10^th^ January 2019 and data was extracted according to the Preferred Reporting Items for Systematic Reviews and Meta-Analyses (PRISMA) guidelines. The quality of the identified studies was assessed per measurement property according to the COnsensus-based Standards for the selection of health Measurements Instruments (COSMIN) checklist.

**Results:**

Seven studies were found evaluating 6 health-related QoL PROMs. Among six, there were 1 disease-specific and 5 generic PROMs. Information regarding important measurement properties was often incomplete. The evidence for the quality of measurement properties was found to be variable, none of the instruments performed sufficient on all measurement properties. Considering results of this review, QOL-OC appeared to have adequate COSMIN measurement properties.

**Conclusion:**

QOL-OC can be implemented in future studies to better understand symptoms and expectations of OC patients and help inform clinicians to formulate treatment strategies as per patients’ needs.

## Introduction

Globally, lip, oral cavity, and pharyngeal cancers have been estimated to be responsible for 529,500 incident cases and 292,300 deaths in 2012, accounting for about 3.8% of all cancer cases and 3.6% of cancer deaths. [1] OC is highly prevalent in Indian subcontinent with tobacco chewing and smoking, betel quid chewing, alcohol consumption and human papilloma virus (HPV) are considered to be the most common risk factors. [2–3] In India, OC is the third most common cancers with (7.2) with high mortality and morbidity rate. [4] As unnoticeable initially, OC usually is diagnosed at later stages carrying 5-year survival rate of only 40–50%. [5,6] Along with manifestations including pain, burning sensation [7], toothache, tooth mobility [8], OC is strongly associated with social and psychological morbidity [9,10] leading to poor quality of life (QoL). [10–13] Despite of several advantages, the available treatment options induces functional impairments such as disabled mastication, deglutition, phonetics and facial disfigurement, consequently again decreasing the post-treatment QoL of these patients.

In recent decades, health-related QoL (HRQoL) has gained an increased importance in clinical practice and research. The HRQoL can be assessed using patient reported outcome measures (PROMs). The PROMs not only reveals patients’ perspectives but also helps to monitor treatment responses. This allows clinicians to elaborate a more exhaustive clinical control of patients and formulate a comprehensive treatment protocol as per individual patient’s needs and expectations. [14] This concept of HRQoL assessment using PROMs is gradually replacing the traditional indicators of health outcomes. [15] Traditionally considered indicators were mainly based on the clinical features and the treatment responses and thus lack the patient’s perspective.

Currently, numerous PROMs have been available in the literature for use in OC patients. [16–20] These include European Organization for Research and Treatment of Cancer Quality of Life Questionnaire Head and Neck-specific module (EORTC QLQ-H&N35), the University of Washington Quality of Life (UWQOL), Functional Assessment of Cancer Therapy- Head and Neck module FACT-H&N (v 4.0), Speech Handicap Index (SHI), Swallowing Quality of Life Questionnaire (SWAL-QOL), Cancer needs questionnaire, short form, head and neck cancer-specific (CNQ-SF-hn) and Oral Cancer Quality-of-life Questionnaire (QOL-OC). However, as they vary significantly with respect to their development and validation, none has clearly been considered as gold standard. Selecting an adequate PROM is a pre-requisite in order to obtain an optimal informative HRQoL data. Since, no review have been conducted systematically to assess the psychometric properties of the validation studies on PROMs for OC patients till date, the present systematic review was designed with the objectives of (i) identifying PROMs used in validation studies involving OC patients and (ii) to determine their measurement properties.

## Materials and methods

This review was performed according to (i) the Preferred Reporting Items for Systematic Reviews and Meta-Analyses (PRISMA) [21] statement (S1 Table), (ii) COnsensus based Standards for the selection of health Measurement INstruments (COSMIN) guidance. [22]

### Search strategy

A comprehensive search was performed on 10^th^ January 2019 on electronic databases: Medline through PubMed, Scopus, Web of Science and CINAHL. A search strategy (S2 and S3 Tables) was developed to identify relevant publications. In addition, we also searched the reference lists of all relevant review papers.

### Selection criteria

All full text original research papers that assessed HRQoL of adult OC patients (≥ 18 years old) using PROM, if the paper was a validation study or determined one or more psychometric properties of a PROM were considered as eligible articles. No restriction was applied on sample size, gender of the participants, country of origin of the study, date and language of the publication. Non-validation studies were excluded. As per Terwee et al. [23] criteria, publications including editorials and case reports were excluded.

### Study selection

The titles and abstracts of the relevant studies were screened by two reviewers (SG and AG) independently. Accordingly, full texts of all potentially eligible papers were retrieved and screened by the same reviewers (SG and AG) independently. Any disagreements were resolved by discussion with third reviewer (SS).

### Data extraction

Full text articles were assessed by two reviewers (SG and AG) again independently and extracted the following predefined data from each article: PROM used, country and language, study population, number of patients, mode of administration, number of domains, scoring methods and recall periods used. Additionally, data required to complete the COSMIN checklist assessment were also extracted.

### Measurement properties

The methodological quality of the included studies was evaluated by the same two reviewers independently using the COSMIN checklist and any disagreements were resolved by discussion with the third reviewer (SS). The checklist covers nine measurement properties: internal consistency, reliability, measurement error, content validity, construct validity, criterion validity, hypothesis testing, structural validity and responsiveness. Each measurement property was scored as per the quality of reporting by the studies and rated as ‘excellent’, ‘good’, ‘fair’ or ‘poor’. An overall score of each study is then determined by the ‘worst score counts’ method. The methodological quality of each study was rated by taking the lowest score (worst score counts method) per domain. For example, if any of the items of the domain reliability was scored ‘poor’, the overall score for regarding the methodological quality of reliability was rated as ‘poor’.

For each PROM, the psychometric results was determined according to Terwee et al. [24] and ranked as ‘+’ positive, ‘?’ indeterminate or ‘-’ negative.

## Results

### Search results

**Fig 1:**
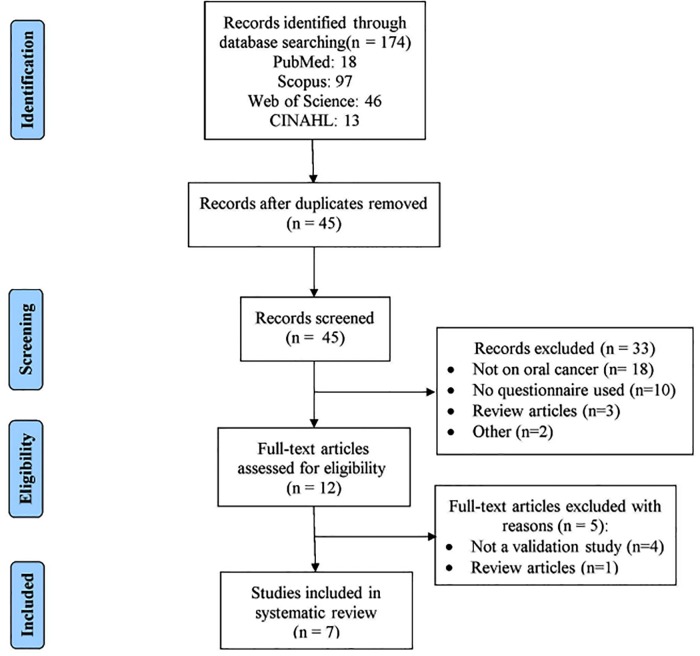
PRISMA flow diagram showing flow of information.

Fig 1 shows the PRISMA flow diagram of the literature search and results. We identified 174 papers from the initial search from different electronic databases (PubMed:18, Web of Science: 46, Scopus:97, CINAHL:13) and 45 remained after excluding duplicates. After screening titles and abstracts for relevance, 33 articles were removed. Full text articles of remaining 12 papers were assessed in depth by two reviewers (SG and AG) for their eligibility, amongst which, five articles were excluded because of non-validation study and review articles. Finally, we included seven papers [16–20, 25,26] that met the inclusion criteria in the present systematic review. All the studies were based on the observations made on the self-administered questionnaire in oral cancer.

### Study characteristics

All the seven studies were conducted on adult OC and oropharyngeal cancer (OPC) patients. In total, 769 patients (range n = 38–213) were recruited. The mean age of the participants was between 53.84 and 62.3 years and a higher proportion of males (55.26% to 93.7%) was reported in all studies, with exception of one study [16] which had higher female participants (61.8%). Total five studies performed the clinical staging, among which 3 studies [16,17,26] had higher proportion of stage IV disease (35.4% to 42%) and remaining 2 studies reported more number of patients with stage II [18] and stage I OC. [20]

### PROMs

Table 1 shows characteristics of all 6 included PROMs, which were developed/validated in OC and OPC patients between 2008 (SHI) and 2018 (QOL-OC). Among six, only one PROMs was disease-specific (QOL-OC) and remaining five were generic (SHI, SWAL-QOL, Chinese version CNQ-SF-hn, Chinese version SHI and FACT-H&N). The number of items included in these PROMs ranged from 29 (QOL-OC) to 44 (SWAL-QOL) and had 2 (SHI) to 10 (SWAL-QOL) HRQoL domains. The scoring methods used by all 6 PROMs contained total scores and subscale scores. The target population of all the PROMs were patients with OC and OPC specifically. An average completion time of three PROMs was reported from 5 minutes (Chinese version CNQ-SF-hn) to 10.4 minutes (QOL-OC). The PROMs included in this review were originally developed/validated in The Netherlands (SHI, SWAL-QOL) [20,25,26], China (Chinese version SHI, QOL-OC) [17,18], Taiwan (Chinese version CNQ-SF-hn) [19] and Malaysia (FACT-H&N) [16].

**Table 1:** Overview of the included studies.

Sr. no.	Author (Year)	Questionnaire	Acronym	Mode of administration	Country/ language	Purpose of the measurement	HRQoL domains	Year of development/validation	Target population	Number of items	Scoring method	Completion time
1.	Rinkel RN et al. (2008) [25]	Speech Handicap Index	SHI	Self-administered	The Netherlands/Dutch	Speech problems of OC, PC patients	S, PSF	2008	Patients with OC, PC	30	Subscale and total scores	NR
2.	Rinkel RN et al. (2009) [26]	Swallowing Quality of Life Questionnaire	SWAL-QOL	Self-administered	The Netherlands/ Dutch	Swallowing outcomes of OC patients	FS, ED, ED, F, B, MH, SF, C, SL, F	2009	Patients with OC, OPC	44	Subscale and total scores	NR
3.	Chen SC et al. (2011) [17]	Cancer needs questionnaire, short form, head and neck cancer-specific	CNQ-SF-hn (Chinese-version)	Self-administered	Taiwan/Chinese	Oral cancer patients’ care needs	PSN, HIN, PDLN,PCSN, ICN, HNDN	2011	Patients with OC	36	Subscale and total scores	5–15 minutes
4.	Doss JG et al. (2011) [16]	Functional Assessment of Cancer Therapy scale 4 (v 4.0)	FACT-H&N (v 4.0)	Self-administered	Malaysia/Malay	HRQoL of OC patients	PWB, SWB EWB, FWB, HN	2011	Patients with OC	43	Subscale and total scores	NR
5.	Li T et al. (2016) [18]	Speech Handicap Index	SHI (Chinese-version)	Self-administered	China/Chinese	Speech problems of OC patients	S, PSF	2016	Patients with OC, OPC	30	Subscale and total scores	NR
6.	Rinkel RN et al. (2015) [20]	Speech Handicap Index and Swallowing Quality of Life Questionnaire	SHI AND SWAL-QOL	Self-administered	The Netherlands/ Dutch	Speech problems & Swallowing outcomes of OC, OPC patients	S, PSF AND FS, ED, ED, F, B, MH, SF, C, SL, F	2015	Patients with OC, OPC	SHI- 30 SWAL-QOL- 44	Subscale and total scores	10 minutes
7.	Nie M et al. (2018) [19]	Oral Cancer Quality-of-life Questionnaire	QOL-OC	Self-administered	China/Chinese	Quality of Life of OC patients	PD, ET, SA, SC, DT	2018	Patients with OC	29	Subscale and total scores	10.4 minutes

**SHI-** S: Speech, PSF: Psychosocial functions

**SWAL-QOL-** FS: Food selection, EDu: eating duration, ED: eating desire, F: fear, B: burden, MH: mental health, SF: social functioning, C: communication, SL: sleep, F: fatigue

**CNQ-SF-hn-** PSN: Psychological needs, HIN: health information needs, PDLN: physical and daily living needs, PCSN: patient care and support needs, ICN: patient care and support needs, HNDN: head and neck disease needs

**FACT-H&N (v 4.0)-** PWB: Physical well-being, SWB: social well-being, EWB: emotional well-being, FWB: functional well-being, HNSC: head & neck

**QOL-OC-** PD: Pain & discomfort, ET: eating, SA: saliva, SC: social contact, DT: diet

OC: Oral cancer, PC: Pharyngeal cancer, OPC: Oro-pharyngeal cancer, HRQoL: Health-related quality of life, NR: Not reported

### Measurement properties

An overview of the measurement properties of all the PROMs is presented in Table 2.

**Table 2:** Rating of measurement properties of the instruments.

Sr. no.	Author (Year)	Measurement instrument	Population	Sample size	Content validity	Construct validity	Internal consistency	Reliability	Absolute measurement error	Responsiveness	Interpretability
1.	Rinkel RN et al. (2008) [25]	SHI	Patients with OC	92	?	+	?	?	NR	NR	NR
2.	Rinkel RN et al. (2009) [26]	SWAL-QOL	Patients with OC	102	?	+	+	?	NR	NR	NR
3.	Chen SC et al. (2011) [17]	CNQ-SF-hn	Patients with OC	206	+	+	+	+	NR	NR	NR
4.	Doss JG et al. (2011) [16]	FACT-H&N (v 4.0)	Patients with OC, OPC	76	+	+	+	?	NR	NR	NR
5.	Li T et al. (2016) [18]	SHI (Chinese-version)	Patients with OC, OPC	42	?	+	?	?	NR	NR	NR
6.	Rinkel RN et al. (2015) [20]	SHI and SWAL-QOL	Patients with OC, PC	38	?	+	?	?	NR	NR	NR
7.	Nie M et al. (2018) [19]	QOL-OC	Patients with OC, OPC	213	+	+	+	+	NR	NR	NR

OC: Oral cancer, PC: Pharyngeal cancer, OPC: Oro-pharyngeal cancer, +: Positive rating, ?: Indeterminate rating, NR: Not reported

#### SHI

The SHI was developed by translating voice handicap index (VHI) into Dutch language. There was no information if the patients were involved in the initial development process. Moreover, it is not clear whether subject experts in the field were involved during this process. Two studies evaluated measurement properties of the SHI. In one study, the SHI was translated into Chinese language and assessed the cross-cultural validity along with other properties. [18] However, other study determined only the construct validity of the SHI. [20]

As it was unclear whether patients and experts were involved in the development process, content validity of the SHI was rated indeterminate. Evidence for excellent construct and structural validity was found. Good cross-cultural validity was reported for Chinese version. Internal consistency and reliability was rated indeterminate for both Dutch and Chinese version due to inadequate sample size.

#### SWAL-QOL

SWAL-QOL was developed by back-translation method into Dutch language. There was no clear information whether patients and experts in the field were involved in development process. Thus, content validity was rated indeterminate. Good evidence was reported construct validity. Internal consistency and reliability was rated indeterminate. One study judged only the construct validity of the SWAL-QOL. [20]

#### CNQ-SF-hn

CNQ-SF-hn was developed by adding few items to previously described CNQ-SF. These newly added items actually reflect unique care needs of head & neck (H&N) cancer patients. The content validity was rated positive because of extensive development process which had involved patients and experts in the field. Factor analysis for structural validity and further internal consistency analysis provided strong evidence for a six-factor structure and thus rated positive. The construct validity was determined by correlating the subscale and total scores of CNQ-SF-hn with other instruments (anxiety, depression, physical performance and QoL). Good evidence was reported for all the domains except for correlation between performance status and interpersonal/communication needs and between QoL and health information needs. Reliability was rated positive.

#### FACT-H&N

FACT-H&N was translated into Malay language for use in Malaysian OC patients. The Malay-translated version was pretested for face and content validity. The FACT-H&N summary scale and subscale showed acceptable construct validity and internal consistency, thus rated positive. No information was provided regarding test-retest reliability and rated indeterminate.

#### QOL-OC

QOL-OC is the only disease-specific PROM developed in China to assess QoL in OC patients. Content validity was rated positive as extensive process involved (included patients, experts in the field and referred previous questionnaires) in the development process. Factor analysis and subsequent internal consistency analysis supported strong evidence for a seven-factor structure. Good evidence was reported for test-retest reliability for summary scales and subscales except for shoulder and neck function scale which showed limited evidence. A moderate to significant correlation was noted between subscale and summary scales of QOL-OC and EORTC QLC-C30. None of the included studies reported information about the measurement properties measurement error responsiveness and interpretability.

Looking at the results of the present review, QOL-OC showed good content and construct validity and the instrument is reliable. The CNQ-SF-hn also demonstrated adequate psychometric properties because of its good internal consistency and reliability. Other instruments had reasonable measurement properties.

## Discussion

As per our first objective, we identified five generic and one disease-specific self-administered PROMs to assess HRQoL in OC patients. Our second objective was to determine the measurement properties of the PROMs. The information obtained from the studies included in the present review revealed that none of the studies reported complete assessment of all the measurement properties of the PROMs studied as per the COSMIN criteria. It is important to note that the quality of majority of the studies was recorded to be fair to poor quality. Moreover, none of the studies have determined measurement error and responsiveness. Surprisingly, one study has evaluated only construct validity of the studied PROMs. Despite the incomplete information in the identified studies, results of this review support disease-specific QOL-OC as a comprehensive PROM to measure HRQoL in OC patients. The CNQ-SF-hn also appeared to be quite suitable for use in OC patients. However, as it was developed/validated by considering H&N cancers, this generic PROM is bit extensive than QOL-OC.

The QOL-OC and CNQ-SF-hn were developed by involving patients and experts in the concerned field. Additionally, these PROMs were pilot tested for the comprehensiveness and intelligibility in patients’ native language, assuring completeness and inclusion of relevant items for OC patients, particularly in QOL-OC. As the PROM was intended for use in H&N cancer patients, CNQ-SF-hn contained additional items, which might not deemed necessary for OC patients. Steinmann et al suggested that lesser length of PROM can be helpful to reduce the time of administration and thus, enhances patients’ compliance. [27] Strong evidence was rated for internal consistency for both these studies as they have performed factor analysis with adequate sample size and provided subscale structures with outcomes per subscale. They have also correlated subscales and summary scales of these PROMs with other used relevant PROMs for OC. These finding of the present review indicates good evidence for content and construct validity of QOL-OC and moderate for CNQ-SF-hn.

Since accurate and reproducible measurements are pre-requisites for an adequate instrument, an acceptable reliability is essential. It was noticed that reliability was studied for QOL-OC and CNQ-SF-hn and demonstrated good evidence. Despite of moderate to good evidence for other PROMs used in OC patients, the evidence for measurement property reliability and measurement error were meager. The inadequate sample size used by the studies resulted in poor quality on reliability. We believe that this striking finding of this review indicates a clear need of re-evaluation of this particular property in future research.

Furthermore, all the studies presented herein intended to quantify the disease burden and its impact using constructs such asHRQoL and severity of symptoms. But, none of the PROMs has determined responsiveness, which can detect changes over time properly. This is an alarming finding of the present review as PROMs are popularly used nowadays as an indicator of quality of care in clinical practice and researches. Although interpretability is not a measurement property but is a meaningful requisite for the applicability of PROMs in research. Since, no study evaluated this, the evidence for interpretability is unknown. However, this does not mean that the PROMs presented herein have poor measurement properties and thus are of poor quality. Other medical fields also showed lack of adequate assessment of all measurement properties in good methodological studies. [28–30] Therefore there is an urgent need of further high quality methodological studies to properly assess and strengthen their measurement properties.

Even though clinicians and researchers can opt for any of the available PROMs as per their choice, the objective and psychometric properties of the PROMs should be taken into consideration while selection. [31] Our review results suggest that generic instruments such SHI, SWAL-QOL, CNQ-SF-hn and FACT-H&N have been used in OC patients. All these PROMs have been widely accepted in the scientific community as being valid, reliable and applicable to a variety of health disparities in H&N cancer patients. Despite the fact that these PROMs may measure burden of OC, they may lack sensitivity in identifying OC-specific problems and can provide misleading outcomes. In addition, SHI and SWAL-QOL are intended to measure speech and swallowing problems of OC patients mainly and limited in other HRQoL domains. Being OC-specific, QOL-OC may be more effective and identify OC-related symptoms and problems such as pain & discomfort, eating problems, oral dryness, diet changes etc. and their impacts on QoL. Since QOL-OC has been developed very recently, it has not been widely investigated. As it has scored more favorably using COSMIN review, we believe that QOL-OC needs to be perceived by the scientific community in the future research to further evaluate its measurement properties in different cultural and language contexts.

### Strengths and limitations

This review is the first attempt to evaluate the methodological quality of the validation studies of PROMs used in OC patients by using PRISMA and COSMIN guidelines. The quality of the included studies was evaluated by two reviewers independently with the help of third reviewer in cases of disagreement. In addition, only those studies were selected in which target population were OC patients. We have searched extensively for papers in different electronic databases without any time and language restrictions to minimize chances of missing relevant publications. Even with the broad search strategy used, we could find only seven validation studies; demonstrating lack of literature in this area. The possibility of the publication bias cannot be ruled out. It could be possible that validation studies with negative results may have never been published. There could be chances that studies have been performed properly but not explained well enough as per COSMIN criteria, thus affecting their quality ratings.

## Conclusion

We identified five generic and one disease-specific PROM used in OC patients. Based on the results of the present review, we agree that QOL-OC may perform better than other available PROMs. As there is decreased QoL in OC patients from diagnosis (threat of cancer development) and even after treatment (uncertainty of successful outcomes), selecting an adequate PROM is a pre-requisite for clinicians. Therefore, we recommend implementation of this PROM in future research for detailed evaluation of patients’ experiences and expectations of OC patients. We also recommend higher quality methodological studies for proper evaluation of measurement properties of other PROMs.

## Supporting information

S1 Table PRISMA checklist. (DOC)

S2 Table Keywords used. (DOCX)

S3 Table Search string results from major databases. (DOCX)
