# Supplementary material for: Measurement properties of oral health related patient reported outcome measures in patients with oral cancer: A systematic review using COSMIN checklist
Source: PLoS One. 2019 Jun 27;14(6):e0218833. doi: 10.1371/journal.pone.0218833 (PMC6597073; doi:10.1371/journal.pone.0218833)
Supplement: S2 Table — (DOCX) [file pone.0218833.s002.docx]

**S3 Table:** Search string results from major databases

| **Database** | **Keyword string** | **Total hits** |
| --- | --- | --- |
| PubMed | ("oral cancer"[MeSH Terms] OR ("oral"[All Fields] AND "cancer"[All Fields] OR "oral squamous cell carcinoma"[All Fields]) OR "oral carcinoma"[All Fields]) AND ("quality of life"[All Fields] OR ("quality of life"[MeSH Terms] OR ("quality"[All Fields] AND "life"[All Fields]) OR "quality of life"[All Fields]) OR QOL [All Fields] OR Validation Studies[PTYP]) | 18 |
| SCOPUS | " oral cancer"  AND  "Quality of life"  AND  "Instrument" | 97 |
| Web of science | " oral cancer"  AND  "Quality of life"  AND “Instrument” | 46 |
| CINAHL | " oral cancer"  AND  "Quality of life"  AND “Instrument” | 13 |
