# Supplementary material for: Measurement properties of oral health related patient reported outcome measures in patients with oral cancer: A systematic review using COSMIN checklist
Source: PLoS One. 2019 Jun 27;14(6):e0218833. doi: 10.1371/journal.pone.0218833 (PMC6597073; doi:10.1371/journal.pone.0218833)
Supplement: S3 Table — (DOCX) [file pone.0218833.s003.docx]

**S2 Table:** Keywords used

| **Topic** | **Keyword** |
| --- | --- |
| Oral cancer | "oral cancer" [MeSH Terms] OR ("oral"[All Fields] AND "cancer"[All Fields] OR "oral squamous cell carcinoma"[All Fields] OR "oral carcinoma"[All Fields] |
| Quality of life | "quality of life"[MeSH Terms] OR ("quality"[All Fields] AND "life"[All Fields]) OR "quality of life"[All Fields] |
